# Constrained hidden Markov models for population-based haplotyping

**DOI:** 10.1186/1471-2105-8-S2-S9

**Published:** 2007-05-03

**Authors:** Niels Landwehr, Taneli Mielikäinen, Lauri Eronen, Hannu Toivonen, Heikki Mannila

**Affiliations:** 1 Machine Learning Lab, Department of Computer Science, Albert-Ludwigs-University Freiburg, Germany; 2 HIIT Basic Research Unit, Department of Computer Science, University of Helsinki, Finland

## Abstract

**Background:**

*Haplotype Reconstruction *is the problem of resolving the hidden phase information in genotype data obtained from laboratory measurements. Solving this problem is an important intermediate step in gene association studies, which seek to uncover the genetic basis of complex diseases. We propose a novel approach for haplotype reconstruction based on constrained hidden Markov models. Models are constructed by incrementally refining and regularizing the structure of a simple generative model for genotype data under Hardy-Weinberg equilibrium.

**Results:**

The proposed method is evaluated on real-world and simulated population data. Results show that it is competitive with other recently proposed methods in terms of reconstruction accuracy, while offering a particularly good trade-off between computational costs and quality of results for large datasets.

**Conclusion:**

Relatively simple probabilistic approaches for haplotype reconstruction based on structured hidden Markov models are competitive with more complex, well-established techniques in this field.

## Background

Analysis of genetic variation in human populations is critical to the understanding of the genetic basis for complex diseases. Most studied differences in DNA are single-nucleotide variations at particular positions in the genome, which are called *single nucleotide polymorphisms *(SNPs). The positions are also called *markers *and the different possible values *alleles*. A *haplotype *is a sequence of SNP alleles along a region of a chromosome, and concisely represents the (variable) genetic information in that region. In the search for DNA sequence variants that are related to common diseases (so-called *gene mapping *studies), haplotype-based approaches have become a central theme [1].

In diploid organisms such as humans there are two *homologous *(i.e., almost identical) copies of each chromosome. Current practical laboratory measurement techniques produce a *genotype *– for *m *markers, a sequence of *m *unordered pairs of alleles. The genotype reveals which two alleles are present at each marker, but not their respective chromosomal origin. In order to obtain haplotypes from genotype data, this hidden phase information needs to be reconstructed. There are two alternative approaches: If family trios are available, most of the ambiguity in the phase can be resolved analytically. If not, population-based computational methods have to be used to estimate the haplotype pair for each genotype. Because trios are more difficult to recruit and more expensive to genotype, population-based approaches are often the only cost-effective method for large-scale studies. Consequently, the study of such techniques has received much attention recently [2,3]. In this paper, we propose and evaluate a novel approach for population-based haplotyping based on constrained hidden Markov models.

### Population-based haplotype reconstruction

A haplotype *h *is a sequence of alleles *h*[*i*] in markers *i *= 1,...,*m*. In most cases, only two alternative alleles occur at an SNP marker, so we can assume that *h *∈ {0, 1}^*m*^. A genotype *g *is a sequence of unordered pairs *g*[*i*] = {hg1
 MathType@MTEF@5@5@+=feaafiart1ev1aaatCvAUfKttLearuWrP9MDH5MBPbIqV92AaeXatLxBI9gBaebbnrfifHhDYfgasaacH8akY=wiFfYdH8Gipec8Eeeu0xXdbba9frFj0=OqFfea0dXdd9vqai=hGuQ8kuc9pgc9s8qqaq=dirpe0xb9q8qiLsFr0=vr0=vr0dc8meaabaqaciaacaGaaeqabaqabeGadaaakeaacqWGObaAdaqhaaWcbaGaem4zaCgabaGaeGymaedaaaaa@3079@[*i*], hg2
 MathType@MTEF@5@5@+=feaafiart1ev1aaatCvAUfKttLearuWrP9MDH5MBPbIqV92AaeXatLxBI9gBaebbnrfifHhDYfgasaacH8akY=wiFfYdH8Gipec8Eeeu0xXdbba9frFj0=OqFfea0dXdd9vqai=hGuQ8kuc9pgc9s8qqaq=dirpe0xb9q8qiLsFr0=vr0=vr0dc8meaabaqaciaacaGaaeqabaqabeGadaaakeaacqWGObaAdaqhaaWcbaGaem4zaCgabaGaeGOmaidaaaaa@307B@[*i*]} of alleles in markers *i *= 1,...,*m*. Hence, *g *∈ {{0, 0}, {1, 1}, {0, 1}}^*m*^. A marker with alleles {0, 0} or {1, 1} is *homozygous *whereas a marker with alleles {0, 1} is *heterozygous*.

#### Problem 1 (haplotype reconstruction)

*Given a multiset *G
 MathType@MTEF@5@5@+=feaafiart1ev1aaatCvAUfKttLearuWrP9MDH5MBPbIqV92AaeXatLxBI9gBamrtHrhAL1wy0L2yHvtyaeHbnfgDOvwBHrxAJfwnaebbnrfifHhDYfgasaacH8akY=wiFfYdH8Gipec8Eeeu0xXdbba9frFj0=OqFfea0dXdd9vqai=hGuQ8kuc9pgc9s8qqaq=dirpe0xb9q8qiLsFr0=vr0=vr0dc8meaabaqaciaacaGaaeqabaWaaeGaeaaakeaaimaacqWFge=raaa@382D@ *of genotypes, find for each g *∈ G
 MathType@MTEF@5@5@+=feaafiart1ev1aaatCvAUfKttLearuWrP9MDH5MBPbIqV92AaeXatLxBI9gBamrtHrhAL1wy0L2yHvtyaeHbnfgDOvwBHrxAJfwnaebbnrfifHhDYfgasaacH8akY=wiFfYdH8Gipec8Eeeu0xXdbba9frFj0=OqFfea0dXdd9vqai=hGuQ8kuc9pgc9s8qqaq=dirpe0xb9q8qiLsFr0=vr0=vr0dc8meaabaqaciaacaGaaeqabaWaaeGaeaaakeaaimaacqWFge=raaa@382D@* the most likely haplotypes *hg1
 MathType@MTEF@5@5@+=feaafiart1ev1aaatCvAUfKttLearuWrP9MDH5MBPbIqV92AaeXatLxBI9gBaebbnrfifHhDYfgasaacH8akY=wiFfYdH8Gipec8Eeeu0xXdbba9frFj0=OqFfea0dXdd9vqai=hGuQ8kuc9pgc9s8qqaq=dirpe0xb9q8qiLsFr0=vr0=vr0dc8meaabaqaciaacaGaaeqabaqabeGadaaakeaacqWGObaAdaqhaaWcbaGaem4zaCgabaGaeGymaedaaaaa@3079@ *and *hg2
 MathType@MTEF@5@5@+=feaafiart1ev1aaatCvAUfKttLearuWrP9MDH5MBPbIqV92AaeXatLxBI9gBaebbnrfifHhDYfgasaacH8akY=wiFfYdH8Gipec8Eeeu0xXdbba9frFj0=OqFfea0dXdd9vqai=hGuQ8kuc9pgc9s8qqaq=dirpe0xb9q8qiLsFr0=vr0=vr0dc8meaabaqaciaacaGaaeqabaqabeGadaaakeaacqWGObaAdaqhaaWcbaGaem4zaCgabaGaeGOmaidaaaaa@307B@* which are a *consistent *reconstruction of g, i.e., g*[*i*] = {hg1
 MathType@MTEF@5@5@+=feaafiart1ev1aaatCvAUfKttLearuWrP9MDH5MBPbIqV92AaeXatLxBI9gBaebbnrfifHhDYfgasaacH8akY=wiFfYdH8Gipec8Eeeu0xXdbba9frFj0=OqFfea0dXdd9vqai=hGuQ8kuc9pgc9s8qqaq=dirpe0xb9q8qiLsFr0=vr0=vr0dc8meaabaqaciaacaGaaeqabaqabeGadaaakeaacqWGObaAdaqhaaWcbaGaem4zaCgabaGaeGymaedaaaaa@3079@[*i*], hg2
 MathType@MTEF@5@5@+=feaafiart1ev1aaatCvAUfKttLearuWrP9MDH5MBPbIqV92AaeXatLxBI9gBaebbnrfifHhDYfgasaacH8akY=wiFfYdH8Gipec8Eeeu0xXdbba9frFj0=OqFfea0dXdd9vqai=hGuQ8kuc9pgc9s8qqaq=dirpe0xb9q8qiLsFr0=vr0=vr0dc8meaabaqaciaacaGaaeqabaqabeGadaaakeaacqWGObaAdaqhaaWcbaGaem4zaCgabaGaeGOmaidaaaaa@307B@[*i*]} *for each i *= 1,...,*m*.

If ℋ
 MathType@MTEF@5@5@+=feaafiart1ev1aaatCvAUfKttLearuWrP9MDH5MBPbIqV92AaeXatLxBI9gBamrtHrhAL1wy0L2yHvtyaeHbnfgDOvwBHrxAJfwnaebbnrfifHhDYfgasaacH8akY=wiFfYdH8Gipec8Eeeu0xXdbba9frFj0=OqFfea0dXdd9vqai=hGuQ8kuc9pgc9s8qqaq=dirpe0xb9q8qiLsFr0=vr0=vr0dc8meaabaqaciaacaGaaeqabaWaaeGaeaaakeaaimaacqWFlecsaaa@3763@ denotes a mapping G
 MathType@MTEF@5@5@+=feaafiart1ev1aaatCvAUfKttLearuWrP9MDH5MBPbIqV92AaeXatLxBI9gBamrtHrhAL1wy0L2yHvtyaeHbnfgDOvwBHrxAJfwnaebbnrfifHhDYfgasaacH8akY=wiFfYdH8Gipec8Eeeu0xXdbba9frFj0=OqFfea0dXdd9vqai=hGuQ8kuc9pgc9s8qqaq=dirpe0xb9q8qiLsFr0=vr0=vr0dc8meaabaqaciaacaGaaeqabaWaaeGaeaaakeaaimaacqWFge=raaa@382D@ → {0, 1}^*m *^× {0, 1}^*m*^, associating each genotype *g *∈ G
 MathType@MTEF@5@5@+=feaafiart1ev1aaatCvAUfKttLearuWrP9MDH5MBPbIqV92AaeXatLxBI9gBamrtHrhAL1wy0L2yHvtyaeHbnfgDOvwBHrxAJfwnaebbnrfifHhDYfgasaacH8akY=wiFfYdH8Gipec8Eeeu0xXdbba9frFj0=OqFfea0dXdd9vqai=hGuQ8kuc9pgc9s8qqaq=dirpe0xb9q8qiLsFr0=vr0=vr0dc8meaabaqaciaacaGaaeqabaWaaeGaeaaakeaaimaacqWFge=raaa@382D@ with a pair ⟨hg1
 MathType@MTEF@5@5@+=feaafiart1ev1aaatCvAUfKttLearuWrP9MDH5MBPbIqV92AaeXatLxBI9gBaebbnrfifHhDYfgasaacH8akY=wiFfYdH8Gipec8Eeeu0xXdbba9frFj0=OqFfea0dXdd9vqai=hGuQ8kuc9pgc9s8qqaq=dirpe0xb9q8qiLsFr0=vr0=vr0dc8meaabaqaciaacaGaaeqabaqabeGadaaakeaacqWGObaAdaqhaaWcbaGaem4zaCgabaGaeGymaedaaaaa@3079@, hg2
 MathType@MTEF@5@5@+=feaafiart1ev1aaatCvAUfKttLearuWrP9MDH5MBPbIqV92AaeXatLxBI9gBaebbnrfifHhDYfgasaacH8akY=wiFfYdH8Gipec8Eeeu0xXdbba9frFj0=OqFfea0dXdd9vqai=hGuQ8kuc9pgc9s8qqaq=dirpe0xb9q8qiLsFr0=vr0=vr0dc8meaabaqaciaacaGaaeqabaqabeGadaaakeaacqWGObaAdaqhaaWcbaGaem4zaCgabaGaeGOmaidaaaaa@307B@⟩ of haplotypes, the goal is to find the ℋ
 MathType@MTEF@5@5@+=feaafiart1ev1aaatCvAUfKttLearuWrP9MDH5MBPbIqV92AaeXatLxBI9gBamrtHrhAL1wy0L2yHvtyaeHbnfgDOvwBHrxAJfwnaebbnrfifHhDYfgasaacH8akY=wiFfYdH8Gipec8Eeeu0xXdbba9frFj0=OqFfea0dXdd9vqai=hGuQ8kuc9pgc9s8qqaq=dirpe0xb9q8qiLsFr0=vr0=vr0dc8meaabaqaciaacaGaaeqabaWaaeGaeaaakeaaimaacqWFlecsaaa@3763@ that maximizes **P**(ℋ
 MathType@MTEF@5@5@+=feaafiart1ev1aaatCvAUfKttLearuWrP9MDH5MBPbIqV92AaeXatLxBI9gBamrtHrhAL1wy0L2yHvtyaeHbnfgDOvwBHrxAJfwnaebbnrfifHhDYfgasaacH8akY=wiFfYdH8Gipec8Eeeu0xXdbba9frFj0=OqFfea0dXdd9vqai=hGuQ8kuc9pgc9s8qqaq=dirpe0xb9q8qiLsFr0=vr0=vr0dc8meaabaqaciaacaGaaeqabaWaaeGaeaaakeaaimaacqWFlecsaaa@3763@ | G
 MathType@MTEF@5@5@+=feaafiart1ev1aaatCvAUfKttLearuWrP9MDH5MBPbIqV92AaeXatLxBI9gBamrtHrhAL1wy0L2yHvtyaeHbnfgDOvwBHrxAJfwnaebbnrfifHhDYfgasaacH8akY=wiFfYdH8Gipec8Eeeu0xXdbba9frFj0=OqFfea0dXdd9vqai=hGuQ8kuc9pgc9s8qqaq=dirpe0xb9q8qiLsFr0=vr0=vr0dc8meaabaqaciaacaGaaeqabaWaaeGaeaaakeaaimaacqWFge=raaa@382D@). It is usually assumed that the sample G
 MathType@MTEF@5@5@+=feaafiart1ev1aaatCvAUfKttLearuWrP9MDH5MBPbIqV92AaeXatLxBI9gBamrtHrhAL1wy0L2yHvtyaeHbnfgDOvwBHrxAJfwnaebbnrfifHhDYfgasaacH8akY=wiFfYdH8Gipec8Eeeu0xXdbba9frFj0=OqFfea0dXdd9vqai=hGuQ8kuc9pgc9s8qqaq=dirpe0xb9q8qiLsFr0=vr0=vr0dc8meaabaqaciaacaGaaeqabaWaaeGaeaaakeaaimaacqWFge=raaa@382D@ is in Hardy-Weinberg equilibrium, i.e., that **P**(⟨hg1
 MathType@MTEF@5@5@+=feaafiart1ev1aaatCvAUfKttLearuWrP9MDH5MBPbIqV92AaeXatLxBI9gBaebbnrfifHhDYfgasaacH8akY=wiFfYdH8Gipec8Eeeu0xXdbba9frFj0=OqFfea0dXdd9vqai=hGuQ8kuc9pgc9s8qqaq=dirpe0xb9q8qiLsFr0=vr0=vr0dc8meaabaqaciaacaGaaeqabaqabeGadaaakeaacqWGObaAdaqhaaWcbaGaem4zaCgabaGaeGymaedaaaaa@3079@, hg2
 MathType@MTEF@5@5@+=feaafiart1ev1aaatCvAUfKttLearuWrP9MDH5MBPbIqV92AaeXatLxBI9gBaebbnrfifHhDYfgasaacH8akY=wiFfYdH8Gipec8Eeeu0xXdbba9frFj0=OqFfea0dXdd9vqai=hGuQ8kuc9pgc9s8qqaq=dirpe0xb9q8qiLsFr0=vr0=vr0dc8meaabaqaciaacaGaaeqabaqabeGadaaakeaacqWGObaAdaqhaaWcbaGaem4zaCgabaGaeGOmaidaaaaa@307B@⟩) = **P**(hg1
 MathType@MTEF@5@5@+=feaafiart1ev1aaatCvAUfKttLearuWrP9MDH5MBPbIqV92AaeXatLxBI9gBaebbnrfifHhDYfgasaacH8akY=wiFfYdH8Gipec8Eeeu0xXdbba9frFj0=OqFfea0dXdd9vqai=hGuQ8kuc9pgc9s8qqaq=dirpe0xb9q8qiLsFr0=vr0=vr0dc8meaabaqaciaacaGaaeqabaqabeGadaaakeaacqWGObaAdaqhaaWcbaGaem4zaCgabaGaeGymaedaaaaa@3079@)**P**(hg2
 MathType@MTEF@5@5@+=feaafiart1ev1aaatCvAUfKttLearuWrP9MDH5MBPbIqV92AaeXatLxBI9gBaebbnrfifHhDYfgasaacH8akY=wiFfYdH8Gipec8Eeeu0xXdbba9frFj0=OqFfea0dXdd9vqai=hGuQ8kuc9pgc9s8qqaq=dirpe0xb9q8qiLsFr0=vr0=vr0dc8meaabaqaciaacaGaaeqabaqabeGadaaakeaacqWGObaAdaqhaaWcbaGaem4zaCgabaGaeGOmaidaaaaa@307B@) for all *g *∈ G
 MathType@MTEF@5@5@+=feaafiart1ev1aaatCvAUfKttLearuWrP9MDH5MBPbIqV92AaeXatLxBI9gBamrtHrhAL1wy0L2yHvtyaeHbnfgDOvwBHrxAJfwnaebbnrfifHhDYfgasaacH8akY=wiFfYdH8Gipec8Eeeu0xXdbba9frFj0=OqFfea0dXdd9vqai=hGuQ8kuc9pgc9s8qqaq=dirpe0xb9q8qiLsFr0=vr0=vr0dc8meaabaqaciaacaGaaeqabaWaaeGaeaaakeaaimaacqWFge=raaa@382D@, and that genotypes are independently sampled from the same distribution. With such assumptions, the likelihood **P**(ℋ
 MathType@MTEF@5@5@+=feaafiart1ev1aaatCvAUfKttLearuWrP9MDH5MBPbIqV92AaeXatLxBI9gBamrtHrhAL1wy0L2yHvtyaeHbnfgDOvwBHrxAJfwnaebbnrfifHhDYfgasaacH8akY=wiFfYdH8Gipec8Eeeu0xXdbba9frFj0=OqFfea0dXdd9vqai=hGuQ8kuc9pgc9s8qqaq=dirpe0xb9q8qiLsFr0=vr0=vr0dc8meaabaqaciaacaGaaeqabaWaaeGaeaaakeaaimaacqWFlecsaaa@3763@ | G
 MathType@MTEF@5@5@+=feaafiart1ev1aaatCvAUfKttLearuWrP9MDH5MBPbIqV92AaeXatLxBI9gBamrtHrhAL1wy0L2yHvtyaeHbnfgDOvwBHrxAJfwnaebbnrfifHhDYfgasaacH8akY=wiFfYdH8Gipec8Eeeu0xXdbba9frFj0=OqFfea0dXdd9vqai=hGuQ8kuc9pgc9s8qqaq=dirpe0xb9q8qiLsFr0=vr0=vr0dc8meaabaqaciaacaGaaeqabaWaaeGaeaaakeaaimaacqWFge=raaa@382D@) of the reconstruction ℋ
 MathType@MTEF@5@5@+=feaafiart1ev1aaatCvAUfKttLearuWrP9MDH5MBPbIqV92AaeXatLxBI9gBamrtHrhAL1wy0L2yHvtyaeHbnfgDOvwBHrxAJfwnaebbnrfifHhDYfgasaacH8akY=wiFfYdH8Gipec8Eeeu0xXdbba9frFj0=OqFfea0dXdd9vqai=hGuQ8kuc9pgc9s8qqaq=dirpe0xb9q8qiLsFr0=vr0=vr0dc8meaabaqaciaacaGaaeqabaWaaeGaeaaakeaaimaacqWFlecsaaa@3763@ given G
 MathType@MTEF@5@5@+=feaafiart1ev1aaatCvAUfKttLearuWrP9MDH5MBPbIqV92AaeXatLxBI9gBamrtHrhAL1wy0L2yHvtyaeHbnfgDOvwBHrxAJfwnaebbnrfifHhDYfgasaacH8akY=wiFfYdH8Gipec8Eeeu0xXdbba9frFj0=OqFfea0dXdd9vqai=hGuQ8kuc9pgc9s8qqaq=dirpe0xb9q8qiLsFr0=vr0=vr0dc8meaabaqaciaacaGaaeqabaWaaeGaeaaakeaaimaacqWFge=raaa@382D@ is proportional to ∏g∈GP(hg1)P(hg2)
MathType@MTEF@5@5@+=feaafiart1ev1aaatCvAUfKttLearuWrP9MDH5MBPbIqV92AaeXatLxBI9gBaebbnrfifHhDYfgasaacH8akY=wiFfYdH8Gipec8Eeeu0xXdbba9frFj0=OqFfea0dXdd9vqai=hGuQ8kuc9pgc9s8qqaq=dirpe0xb9q8qiLsFr0=vr0=vr0dc8meaabaqaciaacaGaaeqabaqabeGadaaakeaadaqeqaqaamrr1ngBPrwtHrhAYaqeguuDJXwAKbstHrhAGq1DVbaceaGae8xgHaLaeiikaGIaemiAaG2aa0baaSqaaiabdEgaNbqaaiabigdaXaaakiabcMcaPiab=LriqjabcIcaOiabdIgaOnaaDaaaleaacqWGNbWzaeaacqaIYaGmaaGccqGGPaqkaSqaaiabdEgaNjabgIGioprtHrhAL1wy0L2yHvtyaeXbnfgDOvwBHrxAJfwnaGqbaiab+zq8hbqab0Gaey4dIunaaaa@53AE@ if the reconstruction is consistent for all *g *∈ G
 MathType@MTEF@5@5@+=feaafiart1ev1aaatCvAUfKttLearuWrP9MDH5MBPbIqV92AaeXatLxBI9gBamrtHrhAL1wy0L2yHvtyaeHbnfgDOvwBHrxAJfwnaebbnrfifHhDYfgasaacH8akY=wiFfYdH8Gipec8Eeeu0xXdbba9frFj0=OqFfea0dXdd9vqai=hGuQ8kuc9pgc9s8qqaq=dirpe0xb9q8qiLsFr0=vr0=vr0dc8meaabaqaciaacaGaaeqabaWaaeGaeaaakeaaimaacqWFge=raaa@382D@, and zero otherwise. In population-based haplotyping, a probabilistic model *λ *for the distribution over haplotypes is estimated from the available genotype information G
 MathType@MTEF@5@5@+=feaafiart1ev1aaatCvAUfKttLearuWrP9MDH5MBPbIqV92AaeXatLxBI9gBamrtHrhAL1wy0L2yHvtyaeHbnfgDOvwBHrxAJfwnaebbnrfifHhDYfgasaacH8akY=wiFfYdH8Gipec8Eeeu0xXdbba9frFj0=OqFfea0dXdd9vqai=hGuQ8kuc9pgc9s8qqaq=dirpe0xb9q8qiLsFr0=vr0=vr0dc8meaabaqaciaacaGaaeqabaWaaeGaeaaakeaaimaacqWFge=raaa@382D@. The distribution estimate **P**(*h *| *λ*) is then used to find the most likely reconstruction ℋ
 MathType@MTEF@5@5@+=feaafiart1ev1aaatCvAUfKttLearuWrP9MDH5MBPbIqV92AaeXatLxBI9gBamrtHrhAL1wy0L2yHvtyaeHbnfgDOvwBHrxAJfwnaebbnrfifHhDYfgasaacH8akY=wiFfYdH8Gipec8Eeeu0xXdbba9frFj0=OqFfea0dXdd9vqai=hGuQ8kuc9pgc9s8qqaq=dirpe0xb9q8qiLsFr0=vr0=vr0dc8meaabaqaciaacaGaaeqabaWaaeGaeaaakeaaimaacqWFlecsaaa@3763@ for G
 MathType@MTEF@5@5@+=feaafiart1ev1aaatCvAUfKttLearuWrP9MDH5MBPbIqV92AaeXatLxBI9gBamrtHrhAL1wy0L2yHvtyaeHbnfgDOvwBHrxAJfwnaebbnrfifHhDYfgasaacH8akY=wiFfYdH8Gipec8Eeeu0xXdbba9frFj0=OqFfea0dXdd9vqai=hGuQ8kuc9pgc9s8qqaq=dirpe0xb9q8qiLsFr0=vr0=vr0dc8meaabaqaciaacaGaaeqabaWaaeGaeaaakeaaimaacqWFge=raaa@382D@ under Hardy-Weinberg equilibrium.

The genetic variation in SNPs is mostly due to two causes: *mutation *and *recombination*. Mutations are relatively rare, they occur with a frequency of about 10^-8^. While SNPs are themselves results of ancient mutations, mutations are usually ignored in statistical haplotype models due to their rarity.

Recombination introduces variability by breaking up the chromosomes of the two parents and reconnecting the resulting segments to form a new and different chromosome for the offspring. Because the probability of a recombination event between two markers is lower if they are near to each other, there is a statistical correlation (so-called *linkage disequilibrium*) between markers which decreases with increasing marker distance. Statistical approaches to haplotype modeling are based on exploiting such patterns of correlation.

## Methods

This section presents the proposed method for haplotype reconstruction. We discuss the statistical model employed and present an incremental algorithm for efficiently learning the model structure from genotype data. Finally, datasets and systems used in the experimental evaluation are described.

### (Hidden) Markov models for haplotyping

**Figure 1:**
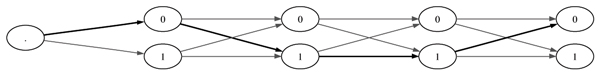
**A Markov model over haplotypes**. The highlighted path encodes the haplotype "0110".

We model the probability distribution on haplotypes by a left-right Markov model *λ *with 2·*m *states, with a state space as shown in Figure 1. A haplotype (of length 4 in the example) is sampled by traversing a path through the model from left to right. The Markov assumption P(h)=∏t=1mPt(h[t]|h[t−1],λ)
MathType@MTEF@5@5@+=feaafiart1ev1aaatCvAUfKttLearuWrP9MDH5MBPbIqV92AaeXatLxBI9gBaebbnrfifHhDYfgasaacH8akY=wiFfYdH8Gipec8Eeeu0xXdbba9frFj0=OqFfea0dXdd9vqai=hGuQ8kuc9pgc9s8qqaq=dirpe0xb9q8qiLsFr0=vr0=vr0dc8meaabaqaciaacaGaaeqabaqabeGadaaakeaatuuDJXwAK1uy0HMmaeHbfv3ySLgzG0uy0HgiuD3BaGabaiab=LriqjabcIcaOiabdIgaOjabcMcaPiabg2da9maaradabaGae8xgHa1aaSbaaSqaaiabdsha0bqabaGccqGGOaakcqWGObaAcqGGBbWwcqWG0baDcqGGDbqxcqGG8baFcqWGObaAcqGGBbWwcqWG0baDcqGHsislcqaIXaqmcqGGDbqxcqGGSaaliiGacqGF7oaBcqGGPaqkaSqaaiabdsha0jabg2da9iabigdaXaqaaiabd2gaTbqdcqGHpis1aaaa@571C@ is motivated by the observation that linkage disequilibrium decreases with increasing marker distance.

Parameters are of the form **P**_*t*_(*h*[*t*] | *h*[*t *- 1], *λ*), the probability of sampling the new allele *h*[*t*] at position *t *after observing the allele *h*[*t *- 1] at position *t *- 1. Note that separate (conditional) allele distributions **P**_*t *_are defined for every sequence position *t *∈ {1,...,*m*}, as linkage disequilibrium patterns will vary for different markers. This also means that the allele encoding at a given marker position, i.e., which allele is represented as '0' and which as '1', does not affect the distributions that can be represented.

**Figure 2:**
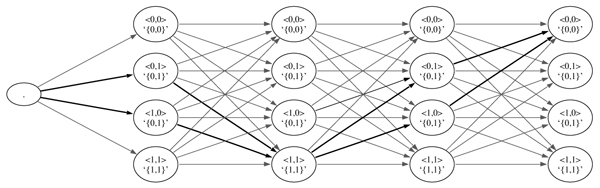
**A hidden Markov model over genotypes**. Possible paths for genotype observation '{0, 1}', '{1, 1}', '{0, 1}', '{0, 0}' are highlighted. The corresponding haplotype pairs are {(0100, 1110), (0110, 1100), (1100, 0110), (1110, 0100)}.

This model is not directly applicable in haplotype reconstruction, because in reality only genotypes are observed whereas the phase information is hidden. The hidden phase information can be modeled by a hidden Markov model *λ' *as shown in Figure 2. A path through this model corresponds to sampling a pair of haplotypes (ordered allele pairs, in angle brackets), while the corresponding genotype (unordered pairs, in curly brackets) is emitted.

To reflect the Hardy-Weinberg equilibrium assumption, constraints have to be placed on transition probabilities. A transition in this model corresponds to independently sampling two new alleles *h*^1^[*t*] and *h*^2^[*t*] at marker *t *based on their respective histories *h*^1^[*t *- 1] and *h*^2^[*t *- 1]. Therefore, the corresponding probability is actually the product of probabilities for sampling *h*^*i*^[*t*] after *h*^*i*^[*t *- 1]:

**P**_*t*_(*h*^1^[*t*], *h*^2^[*t*] | *h*^1^[*t *- 1], *h*^2^[*t *- 1], *λ'*) = **P**_*t*_(*h*^1^[*t*] | *h*^1^[*t *- 1], *λ*)**P**_*t*_(*h*^2^[*t*] | *h*^2^[*t *- 1], *λ*).

In this way, all parameters of *λ' *can be re-expressed as products of parameters of the model *λ *on haplotypes outlined above. Furthermore, *λ' *can be transformed into an equivalent HMM in which these constraints involving products of parameters are replaced with standard parameter tying constraints, which tie parameters in *λ' *to those in *λ*.

An advantage of this approach is that the model *λ' *can be trained directly from genotype data using Baum-Welsh algorithm [4], while implicitly estimating the distribution over haplotypes encoded in *λ*. Furthermore, the most likely reconstruction of a genotype can be directly obtained by the Viterbi algorithm [4]. The presented idea of embedding a model on haplotypes into a model on genotypes in which the genotype phase is the hidden state information, and learning this model using EM, is related to the approaches used in the HIT [5] and fastPHASE [6] systems. In HIT, haplotypes are modeled as recombinations of a set of founder haplotypes, and an instance of the EM algorithm is derived to directly estimate the founders from genotype observations. In fastPHASE, haplotypes are modeled using local clusters, and cluster membership of a haplotype is determined by a hidden Markov model. Again, an instance of the EM algorithm for estimating the clusters directly from genotype data can be derived.

#### Higher-order models and sparse distributions

The main limitation of the model presented so far is that it only takes into account dependencies between adjacent markers. Expressivity can be increased by using a Markov model of order *k *> 1 for the underlying haplotype distribution [7]:

P(h)=∏t=1mPt(h[t]|h[t−k,t−1],λ),
 MathType@MTEF@5@5@+=feaafiart1ev1aaatCvAUfKttLearuWrP9MDH5MBPbIqV92AaeXatLxBI9gBaebbnrfifHhDYfgasaacH8akY=wiFfYdH8Gipec8Eeeu0xXdbba9frFj0=OqFfea0dXdd9vqai=hGuQ8kuc9pgc9s8qqaq=dirpe0xb9q8qiLsFr0=vr0=vr0dc8meaabaqaciaacaGaaeqabaqabeGadaaakeaatuuDJXwAK1uy0HMmaeHbfv3ySLgzG0uy0HgiuD3BaGabaiab=LriqjabcIcaOiabdIgaOjabcMcaPiabg2da9maarahabaGae8xgHa1aaSbaaSqaaiabdsha0bqabaGccqGGOaakcqWGObaAcqGGBbWwcqWG0baDcqGGDbqxcqGG8baFcqWGObaAcqGGBbWwcqWG0baDcqGHsislcqWGRbWAcqGGSaalcqWG0baDcqGHsislcqaIXaqmcqGGDbqxcqGGSaaliiGacqGF7oaBcqGGPaqkcqGGSaalaSqaaiabdsha0jabg2da9iabigdaXaqaaiabd2gaTbqdcqGHpis1aaaa@5CD9@

where *h*[*j*, *i*] is a shorthand for *h*[max{1, *j*}]...*h*[*i*]. Unfortunately, the number of parameters in such a model increases exponentially with the history length *k*. Fortunately, observations on real-world data (e.g., [8]) show that only few conserved haplotype fragments from the set of 2^*k *^possible binary strings of length *k *actually occur. This can be exploited by modeling sparse distributions, where fragment probabilities which are estimated to be very low are set to zero. More precisely, let *p *= **P**_*t*_(*h*[*t*] | *h*[*t *- *k*, *t *- 1]) and define for some small *ε *> 0 a regularized distribution

P^t(h[t]|h[t−k,t−1])={0if p≤ε;1ifp>1−ε;potherwise.
 MathType@MTEF@5@5@+=feaafiart1ev1aaatCvAUfKttLearuWrP9MDH5MBPbIqV92AaeXatLxBI9gBaebbnrfifHhDYfgasaacH8akY=wiFfYdH8Gipec8Eeeu0xXdbba9frFj0=OqFfea0dXdd9vqai=hGuQ8kuc9pgc9s8qqaq=dirpe0xb9q8qiLsFr0=vr0=vr0dc8meaabaqaciaacaGaaeqabaqabeGadaaakeaatuuDJXwAK1uy0HMmaeHbfv3ySLgzG0uy0HgiuD3BaGabaiqb=LriqzaajaWaaSbaaSqaaiabdsha0bqabaGccqGGOaakcqWGObaAcqGGBbWwcqWG0baDcqGGDbqxcqGG8baFcqWGObaAcqGGBbWwcqWG0baDcqGHsislcqWGRbWAcqGGSaalcqWG0baDcqGHsislcqaIXaqmcqGGDbqxcqGGPaqkcqGH9aqpdaGabaqaauaabaaadiaaaeaacqaIWaamaeaaieaacqGFPbqAcqGFMbGzcqqGGaaicqWGWbaCcqGHKjYOiiGacqqF1oqzcqGG7aWoaeaacqaIXaqmaeaacqGFPbqAcqGFMbGztCvAUfeBSjuyZL2yd9gzLbvyNv2CaeXbuLwBLnhiov2DGi1BTfMBaGqbaiaa8bcacqWGWbaCcqGH+aGpcqaIXaqmcqGHsislcqqF1oqzcqGG7aWoaeaacqWGWbaCaeaacqGFVbWBcqGF0baDcqGFObaAcqGFLbqzcqGFYbGCcqGF3bWDcqGFPbqAcqGFZbWCcqGFLbqzcqGGUaGlaaaacaGL7baaaaa@80AB@

If the underlying distribution is sufficiently sparse, P^
 MathType@MTEF@5@5@+=feaafiart1ev1aaatCvAUfKttLearuWrP9MDH5MBPbIqV92AaeXatLxBI9gBaebbnrfifHhDYfgasaacH8akY=wiFfYdH8Gipec8Eeeu0xXdbba9frFj0=OqFfea0dXdd9vqai=hGuQ8kuc9pgc9s8qqaq=dirpe0xb9q8qiLsFr0=vr0=vr0dc8meaabaqaciaacaGaaeqabaqabeGadaaakeaatuuDJXwAK1uy0HMmaeHbfv3ySLgzG0uy0HgiuD3BaGabaiqb=Lriqzaajaaaaa@376D@ can be represented using a relative small number of parameters. The corresponding sparse Markov model structure (in which transitions with probability 0 are removed) will reflect the pattern of conserved haplotype fragments present in the population. How such a sparse model structure can be learned without ever constructing the prohibitively complex distribution **P** will be discussed in the next section.

#### SpaMM: a level-wise learning algorithm

**Algorithm 1 **The level-wise SpaMM learning algorithm.

   Initialize *k *:= 1

   *λ*_1 _:= INITIAL-MODEL()

   *λ*_1 _:= EM-TRAINING(*λ*_1_)

   **repeat**

      *k *:= *k *+ 1

      *λ*_*k *_:= EXTEND-AND-REGULARIZE(*λ*_*k*-1_)

      *λ*_*k *_:= EM-TRAINING(*λ*_*k*_)

   **until ***k *= *k*_*max*_

To construct the sparse order-*k *hidden Markov model, we propose a learning algorithm – called **SpaMM **for **Spa**rse **M**arkov **M**odeling – that iteratively refines hidden Markov models of increasing order (Algorithm 1). More specifically, the idea of SpaMM is to identify conserved fragments using a level-wise search, i.e., by extending short fragments (in low-order models) to longer ones (in high-order models), and is inspired by the well-known Apriori data mining algorithm [9]. The algorithm starts with a first-order Markov model *λ*_1 _on haplotypes where initial transition probabilities are set to P˙
 MathType@MTEF@5@5@+=feaafiart1ev1aaatCvAUfKttLearuWrP9MDH5MBPbIqV92AaeXatLxBI9gBaebbnrfifHhDYfgasaacH8akY=wiFfYdH8Gipec8Eeeu0xXdbba9frFj0=OqFfea0dXdd9vqai=hGuQ8kuc9pgc9s8qqaq=dirpe0xb9q8qiLsFr0=vr0=vr0dc8meaabaqaciaacaGaaeqabaqabeGadaaakeaatuuDJXwAK1uy0HMmaeHbfv3ySLgzG0uy0HgiuD3BaGabaiqb=Lriqzaacaaaaa@3766@_*t*_(*h*[*t*] | *h*[*t *- 1], *λ*_1_) = 0.5 for all *t *∈ {1,...,*m*}, *h*[*t*], *h*[*t *- 1] ∈ {0, 1}. This model can be embedded into a hidden Markov model λ′1
 MathType@MTEF@5@5@+=feaafiart1ev1aaatCvAUfKttLearuWrP9MDH5MBPbIqV92AaeXatLxBI9gBaebbnrfifHhDYfgasaacH8akY=wiFfYdH8Gipec8Eeeu0xXdbba9frFj0=OqFfea0dXdd9vqai=hGuQ8kuc9pgc9s8qqaq=dirpe0xb9q8qiLsFr0=vr0=vr0dc8meaabaqaciaacaGaaeqabaqabeGadaaakeaaiiGacuWF7oaBgaqbamaaBaaaleaacqaIXaqmaeqaaaaa@2F8F@ on genotypes as explained above, and λ′1
 MathType@MTEF@5@5@+=feaafiart1ev1aaatCvAUfKttLearuWrP9MDH5MBPbIqV92AaeXatLxBI9gBaebbnrfifHhDYfgasaacH8akY=wiFfYdH8Gipec8Eeeu0xXdbba9frFj0=OqFfea0dXdd9vqai=hGuQ8kuc9pgc9s8qqaq=dirpe0xb9q8qiLsFr0=vr0=vr0dc8meaabaqaciaacaGaaeqabaqabeGadaaakeaaiiGacuWF7oaBgaqbamaaBaaaleaacqaIXaqmaeqaaaaa@2F8F@ can be trained from the available genotype data using the standard EM algorithm. As parameters in λ′1
 MathType@MTEF@5@5@+=feaafiart1ev1aaatCvAUfKttLearuWrP9MDH5MBPbIqV92AaeXatLxBI9gBaebbnrfifHhDYfgasaacH8akY=wiFfYdH8Gipec8Eeeu0xXdbba9frFj0=OqFfea0dXdd9vqai=hGuQ8kuc9pgc9s8qqaq=dirpe0xb9q8qiLsFr0=vr0=vr0dc8meaabaqaciaacaGaaeqabaqabeGadaaakeaaiiGacuWF7oaBgaqbamaaBaaaleaacqaIXaqmaeqaaaaa@2F8F@ are tied to those in *λ*_1_, this yields new estimates for the parameters **P**_*t*_(*h*[*t*] | *h*[*t *- 1], *λ*_*k*_) in *λ*_*k*_. This training procedure is summarized in the function EM-TRAINING(*λ*_1_).

The function EXTEND-AND-REGULARIZE(*λ*_*k*-1_) takes as input a model of order *k *- 1 and returns a model *λ*_*k *_of order *k*. In *λ*_*k*_, initial transition probabilities are set to

P˙t(h[t]|h[t−k,t−1],λk+1={0if Pt(h[t]|h[t−k+1,t−1],λk)≤ε;1if Pt(h[t]|h[t−k+1,t−1],λk)>1−ε;0.5otherwise,
 MathType@MTEF@5@5@+=feaafiart1ev1aaatCvAUfKttLearuWrP9MDH5MBPbIqV92AaeXatLxBI9gBaebbnrfifHhDYfgasaacH8akY=wiFfYdH8Gipec8Eeeu0xXdbba9frFj0=OqFfea0dXdd9vqai=hGuQ8kuc9pgc9s8qqaq=dirpe0xb9q8qiLsFr0=vr0=vr0dc8meaabaqaciaacaGaaeqabaqabeGadaaakeaatuuDJXwAK1uy0HMmaeHbfv3ySLgzG0uy0HgiuD3BaGabaiqb=LriqzaacaWaaSbaaSqaaiabdsha0bqabaGccqGGOaakcqWGObaAcqGGBbWwcqWG0baDcqGGDbqxcqGG8baFcqWGObaAcqGGBbWwcqWG0baDcqGHsislcqWGRbWAcqGGSaalcqWG0baDcqGHsislcqaIXaqmcqGGDbqxcqGGSaaliiGacqGF7oaBdaWgaaWcbaGaem4AaSMaey4kaSIaeGymaedabeaakiabg2da9maaceaabaqbaeaaamGaaaqaaiabicdaWaqaaGqaaiab9LgaPjab9zgaMjaaykW7cqWFzecudaWgaaWcbaGaemiDaqhabeaakiabcIcaOiabdIgaOjabcUfaBjabdsha0jabc2faDjabcYha8jabdIgaOjabcUfaBjabdsha0jabgkHiTiabdUgaRjabgUcaRiabigdaXiabcYcaSiabdsha0jabgkHiTiabigdaXiabc2faDjabcYcaSiab+T7aSnaaBaaaleaacqWGRbWAaeqaaOGaeiykaKIaeyizImQae4xTduMaei4oaSdabaGaeGymaedabaGae0xAaKMae0NzayMaaGPaVlab=LriqnaaBaaaleaacqWG0baDaeqaaOGaeiikaGIaemiAaGMaei4waSLaemiDaqNaeiyxa0LaeiiFaWNaemiAaGMaei4waSLaemiDaqNaeyOeI0Iaem4AaSMaey4kaSIaeGymaeJaeiilaWIaemiDaqNaeyOeI0IaeGymaeJaeiyxa0LaeiilaWIae43UdW2aaSbaaSqaaiabdUgaRbqabaGccqGGPaqkcqGH+aGpcqaIXaqmcqGHsislcqGF1oqzcqGG7aWoaeaacqaIWaamcqGGUaGlcqaI1aqnaeaacqqFVbWBcqqF0baDcqqFObaAcqqFLbqzcqqFYbGCcqqF3bWDcqqFPbqAcqqFZbWCcqqFLbqzcqGGSaalaaaacaGL7baaaaa@B291@

i.e., transitions are removed if the probability of the transition conditioned on a shorter history is smaller than *ε*. This procedure of iteratively training, extending and regularizing Markov models of increasing order is repeated up to a maximum order *k*_*max*_.

**Figure 3:**
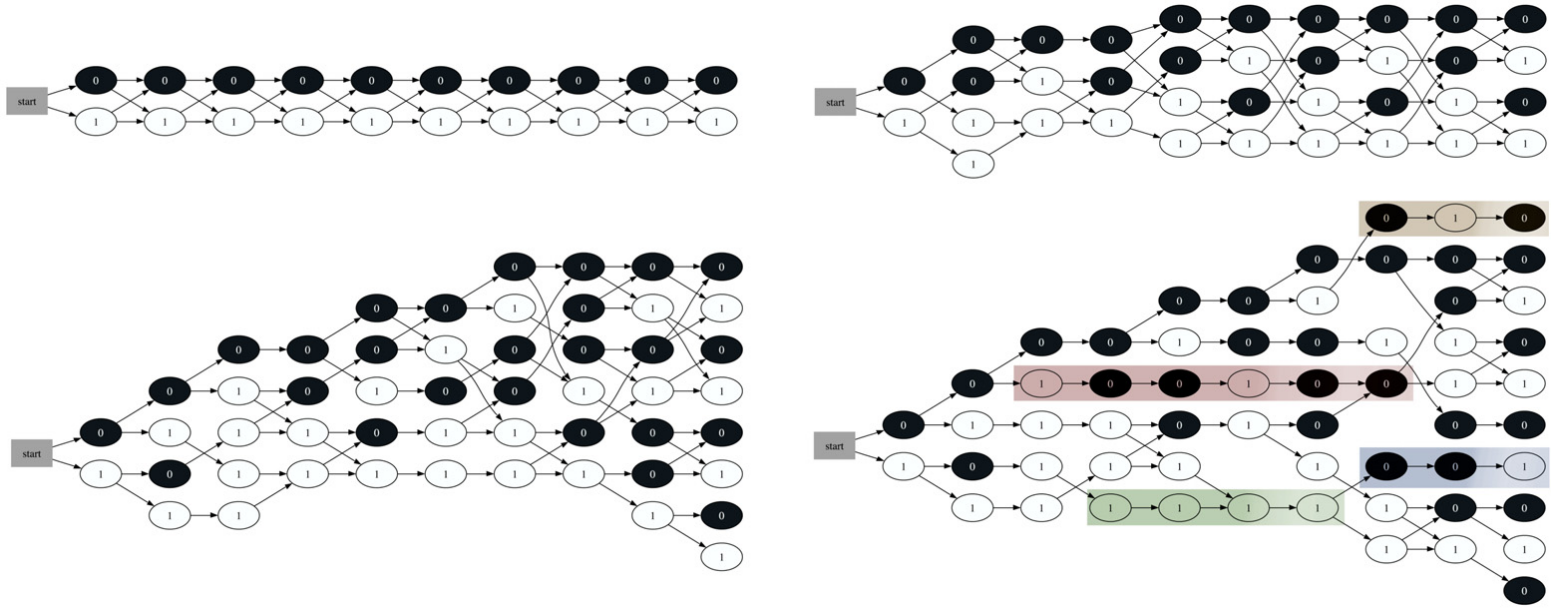
**Visualization of the SpaMM structure learning algorithm**. Sparse models *λ*_1_,...,*λ*_4 _of increasing order learned on the Daly dataset are shown. Black/white nodes encode more frequent/less frequent allele in population. Conserved fragments identified in *λ*_4 _are highlighted.

Figure 3 shows the models learned in the first 4 iterations of the SpaMM algorithm on a real-world dataset. Note how some of the possible transitions are pruned, conserved fragments are isolated and the number of states in the final model is significantly smaller than for a full model of that order. Furthermore, the set of paths through the structure is a concise representation of all haplotypes that have non-zero probability according to the model.

For a given genotype *g*, a reconstructed haplotype pair ⟨hg1
 MathType@MTEF@5@5@+=feaafiart1ev1aaatCvAUfKttLearuWrP9MDH5MBPbIqV92AaeXatLxBI9gBaebbnrfifHhDYfgasaacH8akY=wiFfYdH8Gipec8Eeeu0xXdbba9frFj0=OqFfea0dXdd9vqai=hGuQ8kuc9pgc9s8qqaq=dirpe0xb9q8qiLsFr0=vr0=vr0dc8meaabaqaciaacaGaaeqabaqabeGadaaakeaacqWGObaAdaqhaaWcbaGaem4zaCgabaGaeGymaedaaaaa@3079@, hg2
 MathType@MTEF@5@5@+=feaafiart1ev1aaatCvAUfKttLearuWrP9MDH5MBPbIqV92AaeXatLxBI9gBaebbnrfifHhDYfgasaacH8akY=wiFfYdH8Gipec8Eeeu0xXdbba9frFj0=OqFfea0dXdd9vqai=hGuQ8kuc9pgc9s8qqaq=dirpe0xb9q8qiLsFr0=vr0=vr0dc8meaabaqaciaacaGaaeqabaqabeGadaaakeaacqWGObaAdaqhaaWcbaGaem4zaCgabaGaeGOmaidaaaaa@307B@⟩_*k *_can be obtained from every model *λ*_*k*_. At the same time, the Viterbi algorithm computes **P**(⟨hg1
 MathType@MTEF@5@5@+=feaafiart1ev1aaatCvAUfKttLearuWrP9MDH5MBPbIqV92AaeXatLxBI9gBaebbnrfifHhDYfgasaacH8akY=wiFfYdH8Gipec8Eeeu0xXdbba9frFj0=OqFfea0dXdd9vqai=hGuQ8kuc9pgc9s8qqaq=dirpe0xb9q8qiLsFr0=vr0=vr0dc8meaabaqaciaacaGaaeqabaqabeGadaaakeaacqWGObaAdaqhaaWcbaGaem4zaCgabaGaeGymaedaaaaa@3079@, hg2
 MathType@MTEF@5@5@+=feaafiart1ev1aaatCvAUfKttLearuWrP9MDH5MBPbIqV92AaeXatLxBI9gBaebbnrfifHhDYfgasaacH8akY=wiFfYdH8Gipec8Eeeu0xXdbba9frFj0=OqFfea0dXdd9vqai=hGuQ8kuc9pgc9s8qqaq=dirpe0xb9q8qiLsFr0=vr0=vr0dc8meaabaqaciaacaGaaeqabaqabeGadaaakeaacqWGObaAdaqhaaWcbaGaem4zaCgabaGaeGOmaidaaaaa@307B@⟩_*k *_| *g*, *λ*_*k*_), an estimate of the confidence of the reconstruction. In SpaMM, the reconstruction ⟨hg1
 MathType@MTEF@5@5@+=feaafiart1ev1aaatCvAUfKttLearuWrP9MDH5MBPbIqV92AaeXatLxBI9gBaebbnrfifHhDYfgasaacH8akY=wiFfYdH8Gipec8Eeeu0xXdbba9frFj0=OqFfea0dXdd9vqai=hGuQ8kuc9pgc9s8qqaq=dirpe0xb9q8qiLsFr0=vr0=vr0dc8meaabaqaciaacaGaaeqabaqabeGadaaakeaacqWGObaAdaqhaaWcbaGaem4zaCgabaGaeGymaedaaaaa@3079@, hg2
 MathType@MTEF@5@5@+=feaafiart1ev1aaatCvAUfKttLearuWrP9MDH5MBPbIqV92AaeXatLxBI9gBaebbnrfifHhDYfgasaacH8akY=wiFfYdH8Gipec8Eeeu0xXdbba9frFj0=OqFfea0dXdd9vqai=hGuQ8kuc9pgc9s8qqaq=dirpe0xb9q8qiLsFr0=vr0=vr0dc8meaabaqaciaacaGaaeqabaqabeGadaaakeaacqWGObaAdaqhaaWcbaGaem4zaCgabaGaeGOmaidaaaaa@307B@⟩_k* _with the highest confidence is returned as the final solution:

k∗=arg⁡max⁡k∈{1,...,kmax}P(〈hg1,hg2〉k|g,λk).
 MathType@MTEF@5@5@+=feaafiart1ev1aaatCvAUfKttLearuWrP9MDH5MBPbIqV92AaeXatLxBI9gBaebbnrfifHhDYfgasaacH8akY=wiFfYdH8Gipec8Eeeu0xXdbba9frFj0=OqFfea0dXdd9vqai=hGuQ8kuc9pgc9s8qqaq=dirpe0xb9q8qiLsFr0=vr0=vr0dc8meaabaqaciaacaGaaeqabaqabeGadaaakeaacqWGRbWAcqGHxiIkcqGH9aqpdaWfqaqaaiGbcggaHjabckhaYjabcEgaNjGbc2gaTjabcggaHjabcIha4bWcbaGaem4AaSMaeyicI4Saei4EaSNaeGymaeJaeiilaWIaeiOla4IaeiOla4IaeiOla4IaeiilaWIaem4AaS2aaSbaaWqaaGqaciab=1gaTjab=fgaHjab=Hha4bqabaWccqGG9bqFaeqaamrr1ngBPrwtHrhAYaqeguuDJXwAKbstHrhAGq1DVbaceaGccqGFzecucqGGOaakcqGHPms4cqWGObaAdaqhaaWcbaGaem4zaCgabaGaeGymaedaaOGaeiilaWIaemiAaG2aa0baaSqaaiabdEgaNbqaaiabikdaYaaakiabgQYiXpaaBaaaleaacqWGRbWAaeqaaOGaeiiFaWNaem4zaCMaeiilaWccciGae03UdW2aaSbaaSqaaiabdUgaRbqabaGccqGGPaqkcqGGUaGlaaa@6B66@

The idea of using frequent fragments to build Markov models for haplotypes has also been used in the HaploRec method [7]. In HaploRec, a set of fragments (of any length) that are frequent according to the current model is kept, and updated after each iteration of the EM algorithm.

### Experimental methodology and evaluation

The proposed method was implemented in the SpaMM haplotyping system [10]. We compared its accuracy and computational performance to several other state-of-the art haplotype reconstruction systems: PHASE version 2.1.1 [11], fastPHASE version 1.1 [6], GERBIL as included in GEVALT version 1.0 [12], HIT [5] and HaploRec (variable order Markov model) version 2.0 [13]. All methods were run using their default parameters. The fastPHASE system, which also employs EM for learning a probabilistic model, uses a strategy of averaging results over several random restarts of EM from different initial parameter values. This reduces the variance component of the reconstruction error and alleviates the problem of local minima in EM search. As this is a general technique applicable also to our method, we list results for fastPHASE with averaging (fastPHASE) and without averaging (fastPHASE-NA).

The methods were compared using publicly available real-world datasets, and larger datasets simulated with the Hudson coalescence simulator [14]. As real-world data, we used a collection of datasets from the Yoruba population in Ibadan, Nigeria [1], and the well-known dataset of Daly et al [8], which contains data from a European-derived population. For these datasets, family trios are available, and thus true haplotypes can be inferred analytically. Non-transmitted parental chromosomes of each trio were combined to form additional artificial haplotype pairs. Markers with minor allele frequency of less than 5% and genotypes with more than 15% missing values were removed. Note that if all trio members are heterozygous, the haplotype of the child can not be inferred. In this case, the genotype at this marker position is observed but the marker is ignored when computing the accuracy of the method.

For the Yoruba population, information on 3.8 million SNPs spread over the whole genome is available. We sampled 100 sets of 500 markers each from distinct regions on chromosome 1 (**Yoruba-500**), and from these smaller datasets by taking only the first 20 (**Yoruba-20**) or 100 (**Yoruba-100**) markers for every individual. There are 60 individuals in the dataset after preprocessing, with an average fraction of missing values of 3.6%. For the **Daly **dataset, there is information on 103 markers and 174 individuals available after data preprocessing, and the average fraction of missing values is 8%. Although results on a single dataset are not very meaningful, the Daly dataset was included because it has been used frequently in the literature.

The number of genotyped individuals in these real-world datasets is rather small. For most disease association studies, sample sizes of at least several hundred individuals are needed [15], and we are ultimately interested in haplotyping such larger datasets. Unfortunately, we are not aware of any publicly available real-world datasets of this size, so we have to resort to simulated data. We used the well-known Hudson coalescence simulator [14] to generate 50 artificial datasets, each containing 800 individuals (**Hudson **datasets). The simulator uses the standard Wright-Fisher neutral model of genetic variation with recombination. A chromosomal region of 150 kb was simulated. The probability of mutation in each base pair was set to 10^-8 ^per generation, and the probability of cross-over between adjacent base pairs was set to 10^-8^. These values result in a mutation probability for the entire chromosomal region of *μ *= 0.0015 and cross-over probability of *ρ *= 0.0015. The diploid population size, *N*_0_, was set to the standard 10000, yielding mutation parameter *θ *= 4*N*_0_*μ *= 60, and the recombination parameter *r *= 60. For each data set, a sample of 1600 chromosomes was generated, and these were paired to form 800 genotypes. On average, one simulation produced approximately 493 segregating sites. For each data set, 50 markers were chosen from the segregating sites with minor allele frequency of at least 5%, such that marker spacing was as uniform as possible. The resulting average marker spacing was 3.0 kb. To come as close to the characteristics of real-world data as possible, some alleles were masked (marked as missing) after simulation. More specifically, the missing allele pattern found in the Yoruba datasets was superimposed onto the simulated data, shortening patterns to the size of the target marker map and repeating them as needed for additional individuals.

The accuracy of the reconstructed haplotypes produced by the different methods was measured by normalized switch error. The switch error of a reconstruction is the minimum number of recombinations needed to transform the reconstructed haplotype pair into the true haplotype pair. To normalize, switch errors are summed over all individuals in the dataset and divided by the total number of switch errors that could have been made.

## Results

**Figure 4:**
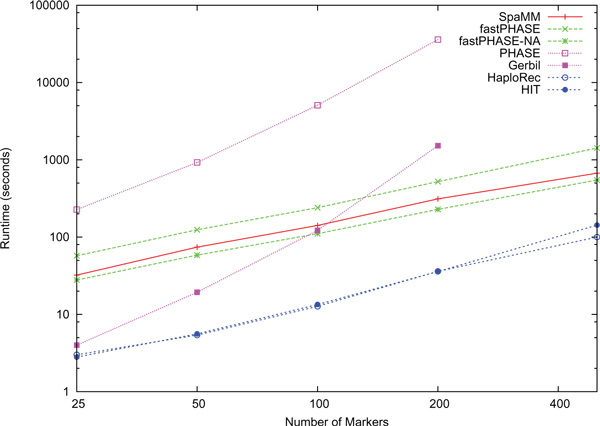
**Runtime as a function of the number of markers**. Average runtime per dataset on Yoruba datasets for marker maps of length 25 to 500 for SpaMM, fastPHASE, fastPHASE-NA, PHASE, Gerbil, HaploRec, and HIT are shown (logarithmic scale). Results are averaged over 10 out of the 100 datasets in the Yoruba collection.

Table 1 shows normalized switch error for all methods on the real-world datasets Yoruba and Daly. For the dataset collections Yoruba-20, Yoruba-100 and Yoruba-500 errors are averaged over the 100 datasets. PHASE and Gerbil did not complete on Yoruba-500 in two weeks (all experiments were run on standard PC hardware with a 3.2 GHz processor and 2 GB of main memory). Overall, the PHASE system achieves highest reconstruction accuracies. After PHASE, fastPHASE with averaging is most accurate, then SpaMM, and then HaploRec. Figure 4 shows the average runtime of the methods for marker maps of different lengths. The most accurate method PHASE is also clearly the slowest. fastPHASE and SpaMM are substantially faster, and HaploRec and HIT very fast. Gerbil is fast for small marker maps but slow for larger ones. For fastPHASE, fastPHASE-NA, HaploRec, SpaMM and HIT, computational costs scale linearly with the length of the marker map, while the increase is superlinear for PHASE and Gerbil, so computational costs quickly become prohibitive for longer maps.

**Figure 5:**
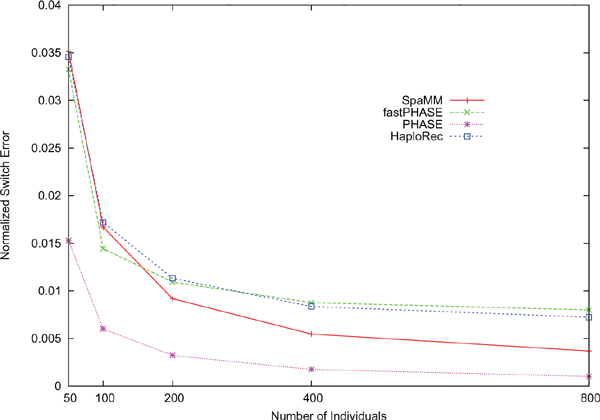
**Reconstruction accuracy as a function of the number of samples available**. Average normalized switch error on the Hudson datasets as a function of the number of individuals for SpaMM, fastPHASE, PHASE and HaploRec is shown. Results are averaged over 50 datasets.

Performance of the systems on larger datasets with up to 800 individuals was evaluated on the 50 simulated Hudson datasets. As for the real-world data, the most accurate methods were PHASE, fastPHASE, SpaMM and HaploRec. Figure 5 shows the normalized switch error of these four methods as a function of the number of individuals (results of Gerbil, fastPHASE-NA, and HIT were significantly worse and are not shown). PHASE was the most accurate method also in this setting, but the relative accuracy of the other three systems depended on the number of individuals in the datasets. While for relatively small numbers of individuals (50–100) fastPHASE outperforms SpaMM and HaploRec, this is reversed for 200 or more individuals.

A problem closely related to haplotype reconstruction is that of genotype imputation. Here, the task is to infer the most likely genotype values (unordered allele pairs) at marker positions where genotype information is missing, based on the observed genotype information. With the exception of HaploRec, all haplotyping systems included in this study can also impute missing genotypes. To test imputation accuracy, between 10% and 40% of all markers were masked randomly, and then the marker values inferred by the systems were compared to the known true marker values. Table 2 shows the accuracy of inferred genotypes for different fractions of masked data on the Yoruba-100 datasets and Table 3 on the simulated Hudson datasets with 400 individuals per dataset. PHASE was too slow to run in this task as its runtime increases significantly in the presence of many missing markers. Evidence from the literature [6] suggests that for this task, fastPHASE outperforms PHASE and is indeed the best method available. In our experiments, on Yoruba-100 fastPHASE is most accurate, SpaMM is slightly less accurate than fastPHASE, but more accurate than any other method (including fastPHASE-NA). On the larger Hudson datasets, SpaMM is significantly more accurate than any other method.

Our experimental results confirm PHASE as the most accurate but also computationally most expensive haplotype reconstruction system [6,11]. If more computational efficiency is required, fastPHASE yields the most accurate reconstructions on small datasets, and SpaMM is preferable for larger datasets. SpaMM also infers missing genotype values with high accuracy. For small datasets, it is second only to fastPHASE; for large datasets, it is substantially more accurate than any other method in our experiments.

The presented method is quite basic: it does not use fine-tuned priors for EM, multiple EM restarts or averaging techniques [5,6], or cross-validates model parameters [6]. Moreover, most statistical models employed in haplotyping are specifically tailored to this problem, and reflect certain assumptions about haplotype structure. For example, the HIT method assumes that there is a limited number of founder haplotypes for a population, and GERBIL assumes block-like haplotype patterns. These systems are only effective if the underlying assumptions are valid. HIT, for instance, was less accurate than PHASE in our study, but has been shown to be competitive with PHASE on population samples from Finland [5], a population isolate for which the assumption of a small number of founders is particularly realistic [16]. Similarly, performance of GERBIL will suffer if haplotypes do not exhibit a block-like structure. In contrast, the sparse higher-order Markov chains used in SpaMM are a general sequence modeling technique. Detailed assumptions about the haplotype structure are replaced by the structure-learning component of the algorithm. The resulting model is rather flexible, and subsumes block-like or mosaic-like haplotype structures (cf. Figure 3). In fact, the proposed approach is not limited to haplotype analysis, and an interesting direction for future work is to apply it also to other sequence modeling tasks.

## Conclusion

We proposed a simple haplotype reconstruction method that is based on iterative refinement and regularization of constrained hidden Markov models (SpaMM). The method was compared against several other state-of-the-art haplotyping systems on real-world genotype datasets with 60–100 individuals and larger simulated datasets with up to 800 individuals. In the experimental study, PHASE was the most accurate, but also computationally most demanding haplotype reconstruction system. fastPHASE and SpaMM are slightly less accurate but much faster, and scale well to long marker maps. The relative performance of these two systems depends on the number of samples available: while fastPHASE is slightly more accurate for small datasets, SpaMM is superior for datasets with several hundred genotype samples. As large datasets are ultimately needed for successful disease association studies, the presented method is a promising alternative to existing approaches.

## Authors' contributions

TM, NL and HM developed the haplotyping method. NL implemented the method and carried out the experiments. LE contributed data to the experimental evaluation. HM and HT coordinated the research. All authors contributed to the preparation of the manuscript.

## Figures and Tables

**Table 1:** Reconstruction accuracy on Yoruba and Daly data.

Method	Yoruba-20	Yoruba-100	Yoruba-500	**Daly**
PHASE	**0.027**	**0.025**	*n.a.*	0.038
fastPHASE	0.033	0.031	**0.034**	**0.027**
SpaMM	0.034	0.037	0.040	0.033
HaploRec	0.036	0.038	0.046	0.034
fastPHASE-NA	0.041	0.060	0.069	0.045
HIT	0.042	0.050	0.055	0.031
GERBIL	0.044	0.051	*n.a*	0.034

Normalized switch error is shown for the Daly dataset, and average normalized switch error over the 100 datasets in the Yoruba-20, Yoruba-100 and Yoruba-500 dataset collections.

**Table 2:** Average error for reconstructing masked genotypes on Yoruba-100.

Method	10%	20%	30%	40%
fastPHASE	**0.045**	**0.052**	**0.062**	**0.075**
SpaMM	0.058	0.066	0.078	0.096
fastPHASE-NA	0.067	0.075	0.089	0.126
HIT	0.070	0.079	0.087	0.098
GERBIL	0.073	0.091	0.110	0.136

From 10% to 40% of all genotypes were masked randomly. Results are averaged over 100 datasets.

**Table 3:** Average error for reconstructing masked genotypes on Hudson.

Method	10%	20%	30%	40%
fastPHASE	0.035	0.041	0.051	0.063
SpaMM	**0.017**	**0.023**	**0.034**	**0.052**
fastPHASE-NA	0.056	0.062	0.074	0.087
HIT	0.081	0.093	0.108	0.127
GERBIL	0.102	0.122	0.148	0.169

From 10% to 40% of all genotypes were masked randomly. Results are averaged over 50 datasets.
